# Porcine Reproductive and Respiratory Syndrome Virus Induces IL-1**β** Production Depending on TLR4/MyD88 Pathway and NLRP3 Inflammasome in Primary Porcine Alveolar Macrophages

**DOI:** 10.1155/2014/403515

**Published:** 2014-05-21

**Authors:** Jing Bi, Shuang Song, Liurong Fang, Dang Wang, Huiyuan Jing, Li Gao, Yidong Cai, Rui Luo, Huanchun Chen, Shaobo Xiao

**Affiliations:** ^1^State Key Laboratory of Agricultural Microbiology, College of Veterinary Medicine, Huazhong Agricultural University, Wuhan 430070, China; ^2^Department of Immunology and Aetology, College of Basic Medicine, Hubei University of Chinese Medicine, Wuhan 430065, China; ^3^Laboratory of Infectious Diseases, College of Veterinary Medicine, Huazhong Agricultural University, 1 Shizishan Street, Wuhan 430070, China

## Abstract

Porcine reproductive and respiratory syndrome virus (PRRSV) is an *Arterivirus* that has been devastating the swine industry worldwide since the late 1980s. Previous studies have reported that PRRSV infection induced the production of IL-1**β**. However, the cellular sensors and signaling pathways involved in this process have not been elucidated yet. Here, we studied the mechanisms responsible for the production of IL-1**β** in response to highly pathogenic PRRSV. Upon PRRSV infection of primary porcine alveolar macrophages, both mRNA expression and secretion of IL-1**β** were significantly increased in a time- and dose-dependent manner. We also investigated the role of several pattern-recognition receptors and adaptor molecules in this response and showed that the TLR4/MyD88 pathway and its downstream signaling molecules, NF-**κ**B, ERK1/2, and p38 MAPKs, were involved in IL-1**β** production during PRRSV infection. Treatment with specific inhibitors or siRNA knockdown assays demonstrated that components of the NLRP3 inflammasome were crucial for IL-1**β** secretion but not for IL-1**β** mRNA expression. Furthermore, TLR4/MyD88/NF-**κ**B signaling pathway was involved in PRRSV-induced expression of NLRP3 inflammasome components. Together, our results deciphered the pathways leading from recognition of PRRSV to the production and release of IL-1**β**, providing a deeper knowledge of the mechanisms of PRRSV-induced inflammation responses.

## 1. Introduction

IL-1*β*, a potent proinflammatory cytokine produced predominantly by monocytes, macrophages, and lymphocytes, plays a central role in regulating immune and inflammatory responses [1, 2]. Production and release of IL-1*β* are induced by a wide variety of stimuli, including microbial infection and tissue injury. Unlike other proinflammatory cytokines, IL-1*β* lacks a signal peptide. The production and secretion of biologically active IL-1*β* involves at least two separate signaling cascades [3, 4]. In the first cascade, pattern recognition receptors (PRRs) in host cells detect microorganisms and induce transcription of an inactive 31-kDa precursor, termed pro-IL-1*β*. In the second signaling cascade, the formation of a large multimeric protein complex known as the inflammasome activates the intracellular cysteine protease caspase 1 and subsequently cleaves pro-IL-1*β* into a 17-kDa mature IL-1*β* [5]. The inflammasome complex is typically composed of three components: (i) a nucleotide-binding oligomerization domain (NOD-) like receptor (NLR); (ii) apoptosis-associated speck-like protein containing a CARD (ASC); and (iii) caspase 1 [6]. Several inflammasomes have been described, of which the NLRP3 inflammasome is the best characterized one [7–9]. NLRP3 contains a C-terminal leucine-rich repeat domain, a central nucleotide binding domain, and an N-terminal PYD. The PYD domain of NLRP3 recruits ASC via a homotypic interaction with the PYD domain. Oligomerization of NLRP3/ASC leads to the recruitment of procaspase 1 to generate enzymatically active caspase 1 which, in turn, processes IL-1*β* [9].

A large number of viruses have been reported to induce the production of IL-1*β* in infected cells [10]. However, different viruses induce the expression of pro-IL-1*β* and process it to its mature form through different signaling pathways and signaling molecules. For example, hepatitis C virus induces IL-1*β* transcription through the TLR7-mediated MyD88 signaling pathway and activates the NLRP3 inflammasome to promote IL-1*β* secretion [11]. Vaccinia virus and murine cytomegalovirus infection stimulate the formation of the absent in melanoma 2 (AIM2) inflammasome to activate caspase 1 the maturation of IL-1*β* [12]. Respiratory syncytial virus (RSV) is mainly recognized by TLR2 and TLR4 to induce IL-1*β* transcription through the NF-*κ*B signaling pathway, and then the NLRP3 inflammasome processes pro-IL-1*β* to its active form [13].

Porcine reproductive and respiratory syndrome (PRRS) is an economically important swine disease characterized by severe reproductive failure in sows and respiratory distress in piglets and growing pigs [14, 15]. The causative agent, PRRSV, is a single-stranded positive-sense RNA virus classified within the family Arteriviridae [16]. Since its emergence in the late 1980s, PRRS has been a threat to the global swine industry, causing high economic losses [17]. PRRSV infection in swine can cause severe interstitial pneumonia [18], suggesting that the inflammatory response plays an important role in infection and pathogenesis of PRRSV. Indeed, Thanawongnuwech et al. reported that the expressions of IL-1*β*, IL-8, and TNF-*α* were significantly elevated in PRRSV-infected pigs [19], while Qiao et al. found that IL-1*β* and TNF-*α* were significantly upregulated in PAMs infected with the highly pathogenic PRRSV strain HN07-1 compared to the classical PRRSV strain BJ-4 [20]. Furthermore, Lunney et al. found that serums IL-1*β* and IL-8 levels significantly correlated with persistent infection of PRRSV [21]. These findings indicated that PRRSV infection significantly induces IL-1*β* expression, and IL-1*β* is closely related to PRRSV pathogenesis.

Although it is clear that PRRSV infection results in IL-1*β* production* in vivo* and* in vitro*, the mechanism of IL-1*β* processing has not been elucidated yet. In this study, we analyzed the underlying mechanisms utilized by porcine alveolar macrophages (PAMs), the target cells of PRRSV infection* in vivo*, to detect PRRSV and induce IL-1*β* expression. Our results revealed that PRRSV-activated IL-1*β* production depended on the TLR4/MyD88 pathway and downstream signaling molecules NF-*κ*B, ERK1/2, and p38 MAPKs. Moreover, we found that the NLRP3/ASC/caspase 1 inflammasome is required for IL-1*β* secretion but not for IL-1*β* transcription during PRRSV infection.

## 2. Materials and Methods

### 2.1. Virus and Cells

PRRSV strain WUH3 [22], originally isolated from the brains of pigs with “high fever syndrome” in China in 2006 and identified as a highly pathogenic type 2 (North American) PRRSV, was used in this study. Primary PAMs were obtained by postmortem lung lavage of 4-week-old pigs from a PRRSV-negative herd as described previously [23]. The animals were housed and handled under conditions approved by the Huazhong Agricultural University Animal Ethics Committee (approval number: HZAU-AEC-30102922). The isolated PAMs were cultured in RPMI-1640 medium (Life Technology, NY, USA). Marc-145 cells, a monkey kidney cell line highly permissive for PRRSV infection, were cultured in DMEM with 10% heated-inactivated FBS, 0.25 *μ*g/mL Fungizone, 100 U/mL penicillin, 10 *μ*g/mL streptomycin sulfate, and 5 *μ*g/mL gentamicin in a humidified 37°C, 5% CO_2_ incubator. PRRSV was amplified in PAMs and viral titers were determined by titration on Marc-145 cells.

### 2.2. Reagents and Antibodies

LPS, polymyxin B (PMB), MAPK inhibitors (SP600125, SB202190, and U0126), NF-*κ*B inhibitor (BAY11-7082), and caspase 1 inhibitor (Boc-D-CMK) were purchased from Sigma-Aldrich (St Louis, MO, USA). SP600125, SB202190, U0126, BAY11-7082, and Boc-D-CMK were dissolved in DMSO and APDC were dissolved in water prior to use. Antibodies specific for p38, ERK1/2, and their phosphorylated forms (p-p38 and p-ERK1/2) were purchased from Cell Signaling Technology (Beverly, MA, USA). Antibody against porcine IL-1*β* was obtained from R&D (Minneapolis, MN, USA). The anti-*β*-actin antibody, HRP-conjugated goat anti-mouse IgG, goat anti-rabbit IgG, and donkey anti-goat IgG were purchased from Sigma-Aldrich (St Louis, MO, USA). A mAb A2F1 used for detection of PRRSV nonstructural protein 2 (Nsp2) was produced from hybridoma cells derived from Sp2/0 myeloma cells and spleen cells of BALB/c mice immunized with recombinant Nsp2 protein of PRRSV strain WUH3 [24].

### 2.3. Viral Plaque Assay

Plaque assay was essentially performed as described previously [25]. Briefly, 95% confluent Marc-145 cells grown in six-well tissue culture plates were infected for 1 h with 10-fold serial dilutions (1000 *μ*L) of PRRSV-containing samples. After three washes with PBS (pH 7.4), cells were overlaid with 1.8% (w/v) Bacto agar mixed 1 : 1 with 2×DMEM containing 0.05 mg/mL neutral red. Plaques were counted 4 days postinfection. The average plaque number and standard deviations were calculated from three independent experiments. Virus titers were expressed as PFU/mL.

### 2.4. Immunofluorescence Assay (IFA)

The infected PAMs were fixed, permeabilized, and subsequently inoculated with the mAb A2F1 against PRRSV Nsp2, followed by immunostaining with a FITC-anti-mouse IgG (Sigma, St. Louis, MO). Cellular nuclei were counterstained with 1 *μ*g/mL of DAPI for 5 min. After washing with PBS, cells were examined under an LSM 510 metaconfocal laser scanning microscope (Carl Zeiss, Göttingen, Germany).

### 2.5. Western Blotting

Cells were lysed in 2% SDS protein sample buffer and subjected to 12% acrylamide SDS-PAGE. The separated proteins were electroblotted onto a nitrocellulose membrane. Western blotting was carried out with the indicated primary antibody and HRP-conjugated antibody. Signals were developed using Supersignal West Pico Luminol kit (Thermo). *β*-Actin was detected with an anti-*β*-actin mAb to demonstrate equal protein sample loading.

### 2.6. siRNA and Cell Transfection

The siRNAs targeting the porcine RIG-I, TLR1, TLR2, TLR3, TLR4, TLR5, TLR6, TLR7, TLR8, TLR9, MyD88, TRIF, NLRP3, and ASC were synthesized by GenePharma (China) and the sequences are listed in Table  S1 available online at http://dx.doi.org/10.1155/2014/403515 in Supplementary Materials. Transient transfection of siRNA was performed using Lipofectamine 2000 (Invitrogen) according to the manufacturer's instructions. The amount of siRNA used for transfection was optimized in preliminary experiments and no significant cellular toxicity was observed.

### 2.7. RNA Extraction and Real-Time RT-PCR

Total cellular RNA was extracted from the treated PAMs using an RNAprep pure cell kit (Qiagen, Valencia, CA, USA). RNA (1 *μ*g) was reverse transcribed in a 20 *μ*L reaction mixture. The cDNA product was amplified in a 20 *μ*L reaction mixture containing SYBR Green real-time PCR Master Mix (Roche, Basel, Switzerland). Gene-specific primers for real-time RT-PCR are listed in Table  S2 (Supplementary Materials). Each cDNA sample was performed in triplicate. PCR amplifications were performed using a Roche LightCycler 480 real-time System (Roche, Basel, Switzerland). Gene expression was measured as a relative quantity as described previously [26].

### 2.8. Measurement of the Secreted IL-1*β* Protein

The secreted IL-1*β* in cell supernatants was measured using a commercial sandwich ELISA kit (R&D) according to the manufacturer's instructions.

### 2.9. Statistical Analyses

All experiments were performed at least three times. Data are presented as mean ± standard deviation (SD). A *P* value less than 0.05 was considered significant and a *P* value less than 0.01 was considered highly significant.

## 3. Results

### 3.1. Infection Kinetics of the Highly Pathogenic PRRSV Strain WUH3 in PAMs

**Figure 1:**
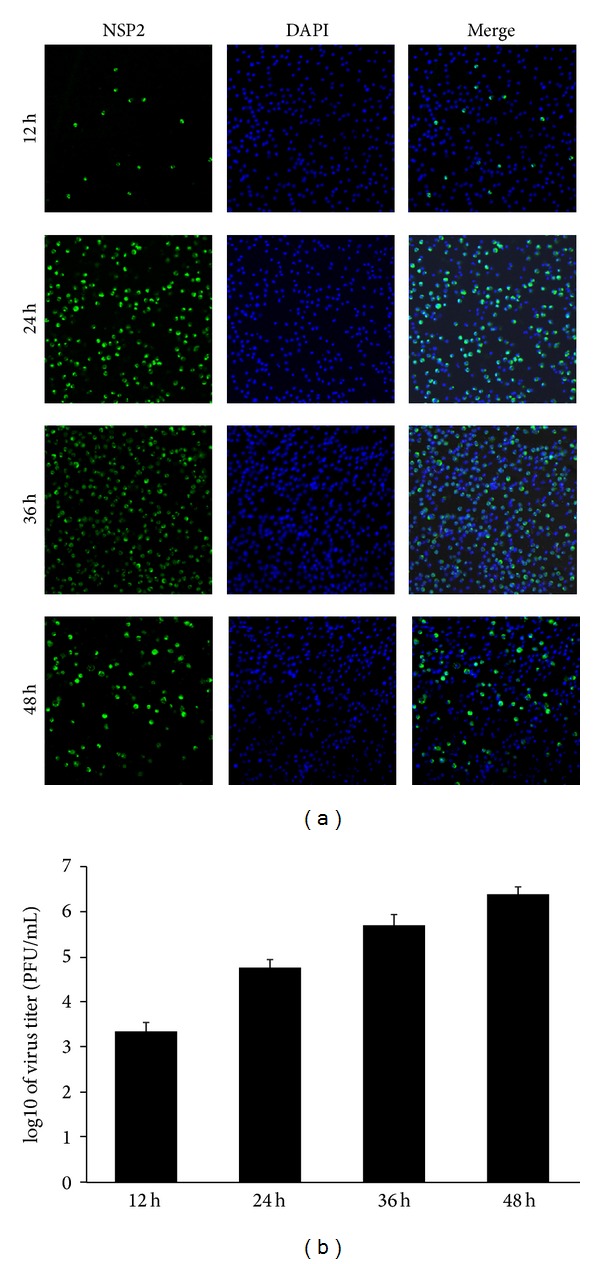
Infection kinetics of the highly pathogenic PRRSV strain WUH3 in PAMs. (a) PAMs were infected with the adapted PRRSV (3rd passages) at an MOI of 0.1. Cells were fixed and permeabilized in cold methanol at different time point (12, 24, 36, and 48 h) postinfection. Immunofluorescence assays were performed to analyze the replication of PRRSV by detecting the nonstructural protein Nsp2 (green fluorescence). DAPI (4′,6-diamidino-2-phenylindole) was used to stain the nuclei. (b) PAMs were infected with the adapted PRRSV at a MOI of 0.1. Supernatants were collected at different time point (12, 24, 36, and 48 h) postinfection for plaque assay to determine viral titers.

The highly pathogenic PRRSV strain WUH3 used in this study was isolated in Marc-145 cells [22], a monkey kidney cell line highly permissive for PRRSV infection* in vitro*. Because PAMs are the target cells of PRRSV infection* in vivo*, PRRSV strain WUH3 was adapted in PAMs for three passages. The infection kinetics of the adapted PRRSV in PAMs were measured by indirect IFA with mAb against PRRSV Nsp2 and plaque assay to titrate virus contained in the supernatant of infected cells. At 24 h postinfection, nearly 50% PAMs were infected and the infected cells became much more at 36 h postinfection. Visible cytopathic effect (CPE) appeared at 48 h postinfection (Figure 1(a)). Plaque assays also showed that the viral titers gradually increased with the progress of PRRSV infection (Figure 1(b)), reaching 10^6.39^ PFU/mL at 48 h postinfection, indicating that the adapted PRRSV can efficiently infect PAMs and spread to neighboring cells. Consequently, the PAMs-adapted PRRSV was used for subsequent experiments.

### 3.2. PRRSV Infection Induces IL-1*β* mRNA Expression and Release in PAMs

**Figure 2:**
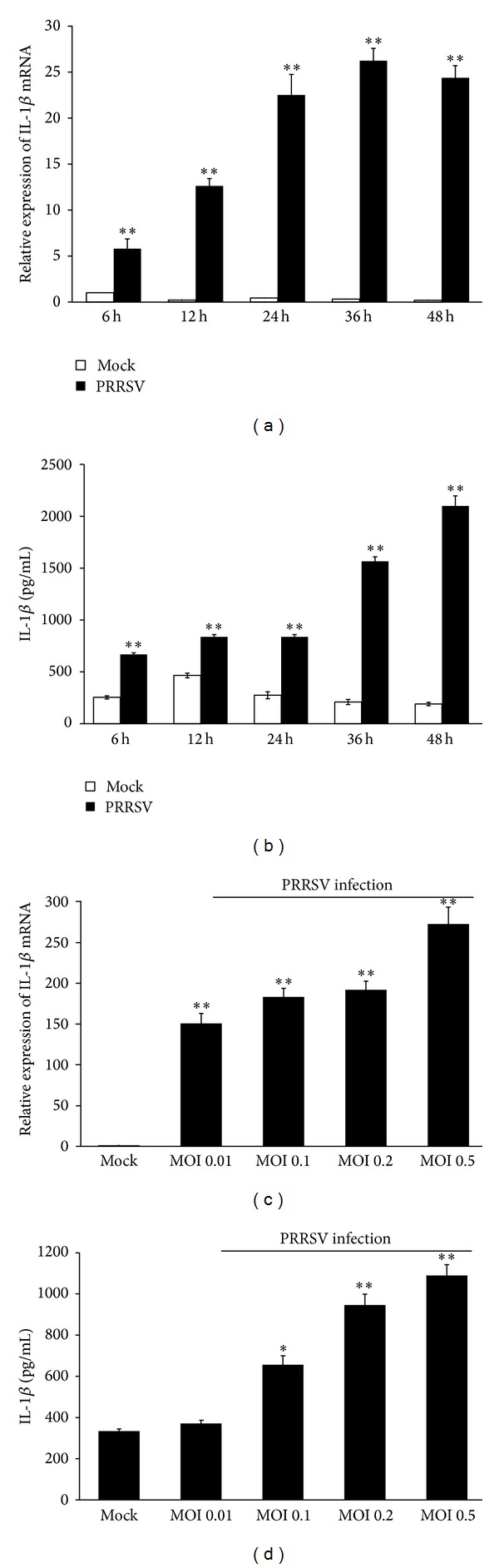
PRRSV infection increases IL-1*β* mRNA expression and secretion in PAMs. (a, b) PAMs were mock infected or infected with PRRSV at a MOI of 0.1. Cells and supernatants were collected separately at the indicated time points and subjected to real-time RT-PCR (a) and ELISA (b) to analyze the expression of IL-1*β*. (c, d) PAMs were infected with PRRSV at different doses (0.01, 0.1, 0.2, and 0.5 MOI). Cells and supernatants were collected separately at 36 h postinfection for analysis by IL-1*β*-specific real-time RT-PCR (c) and ELISA (d). The mock-infected cells were used as negative controls. *P < 0.05 and **P < 0.01 compared with the mock-infected cells.

Previous studies revealed that PRRSV infection induces porcine IL-1*β* expression* in vivo* and* in vitro* [19–21, 27] and the highly pathogenic PRRSV, which has emerged in China and South East Asia since 2006 [28, 29], induced stronger IL-1*β* than the classical PRRSV strains [20]. To investigate the expression kinetics of IL-1*β* after PRRSV infection, primary PAMs were infected with the PAMs-adapted PRRSV strain WUH3 at a MOI of 0.1. Real-time RT-PCR and ELISA were performed to detect IL-1*β* mRNA in cells and the secreted IL-1*β* protein in supernatants, respectively. As shown in Figure 2(a), PAMs infected with PRRSV exhibited significantly increased expression of IL-1*β* mRNA at 6 h postinfection and increased at a steady-state level, with maximal production at 36 h postinfection. Also, significantly increased IL-1*β* release was detected upon PRRSV infection, and the secreted IL-1*β* was further increased as the infection progressed, peaking at later time points (48 h) (Figure 2(b)). When PAMs were infected with PRRSV at increasing MOIs, IL-1*β* mRNA expression and secretion were increased in a dose-dependent manner (Figures 2(c) and 2(d)).

### 3.3. PRRSV-Induced IL-1*β* Production Depends on TLR4-MyD88 Signaling

**Figure 3:**
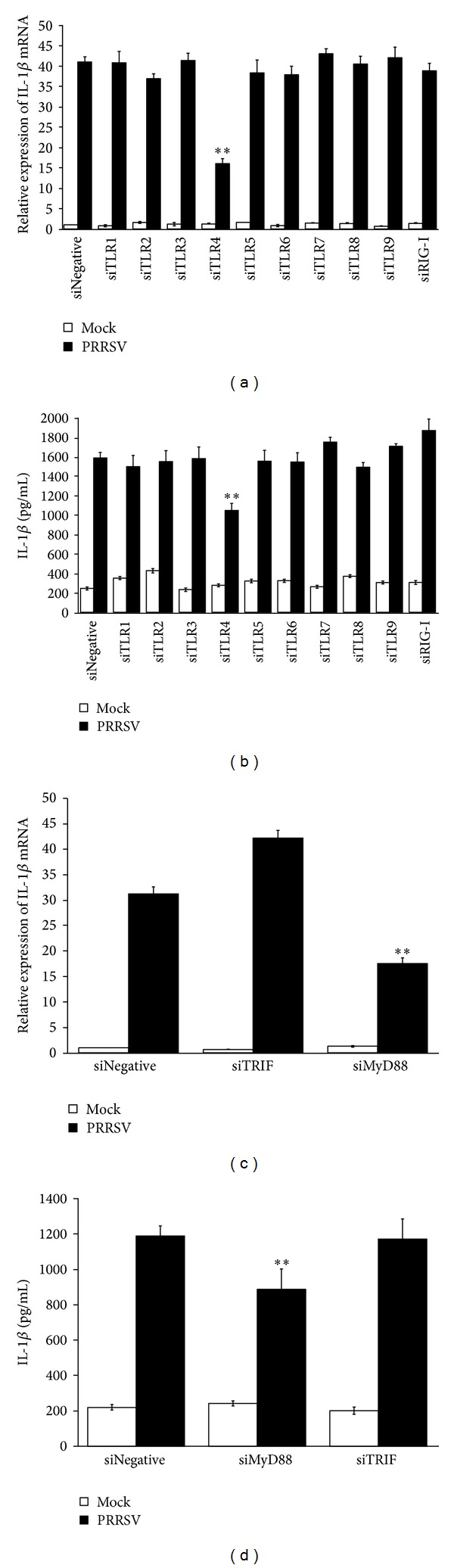
PRRSV-induced IL-1*β* production depends on TLR4-MyD88 pathway. (a, b) PAMs were transfected with 80 nM of psiNegative, psiRIG-I, and psiTLR1-9, respectively. At 24 h after transfection, cells were mock infected or infected with PRRSV at a MOI of 0.1. The cells and supernatants were harvested separately 36 h later and subjected to porcine IL-1*β*-specific real-time RT-PCR (a) and ELISA (b), respectively. (c, d) PAMs were transfected with 80 nM of psiMyD88 and psiTRIF, respectively. At 24 h after transfection, cells were mock infected or infected with PRRSV at a MOI of 0.1. The cells and supernatants were harvested separately 36 h later and analyzed by IL-1*β*-specific real-time RT-PCR (c) and ELISA (d), respectively. *P < 0.05  and **P < 0.01 compared with cells transfected with psiNegative followed by PRRSV infection.

**Figure 4:**
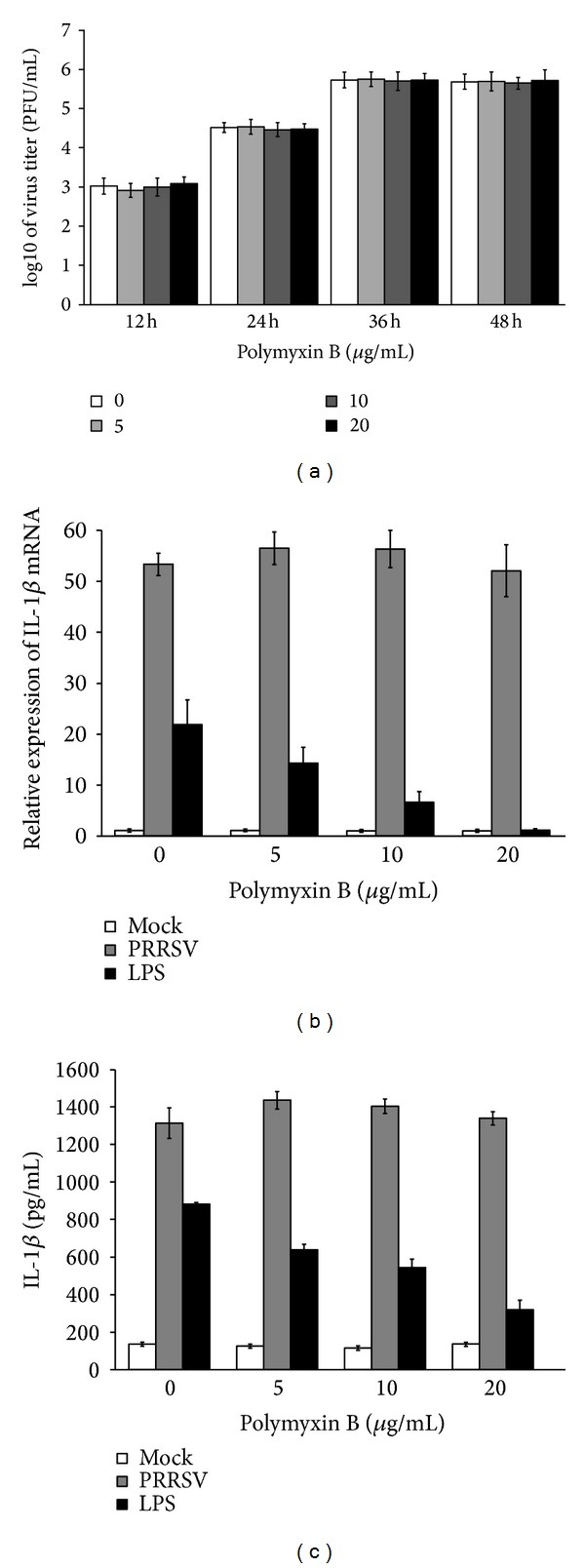
PMB has no effect on PRRSV proliferation or PRRSV-induced IL-1*β* production. (a) PAMs were infected with the adapted PRRSV at a MOI of 0.1, followed by treatment with polymyxin B (0, 5, 10, and 20 *μ*g/mL) in the absence of serum, for 36 h. Supernatants were collected at different time point (12, 24, 36, and 48 h) postinfection for plaque assay to determine viral titers. (b, c) PAMs were infected with the adapted PRRSV at a MOI of 0.1 or treated with LPS (1 *μ*g/mL), followed by treatment with polymyxin B (0, 5, 10, and 20 *μ*g/mL) in the absence of serum, for 36 h. The cells and supernatants were then harvested separately and analyzed by real-time RT-PCR and ELISA, respectively.

During virus invasion, the “first signal” results from the activation of PRRs by pathogen associated molecular patterns (PAMPs) leading to transcription and translation of pro-IL-1*β* [30]. TLRs and RIG-I like receptors (RLRs) are the two main types of PRRs involved in the induction of the innate immune response against RNA viruses [31]. To date, at least 9 members of the porcine TLR family have been identified. In order to investigate the role of TLR and RLR signaling pathways in PRRSV-induced IL-1*β* production, specific siRNA targeting RIG-I or each TLR was designed and synthesized. The knockdown efficiency of siRNA against these receptors was confirmed by real-time PCR (Figure  S1A-J) (Supplementary Materials). Next, PAMs were transfected with specific siRNAs or negative control siRNA (psiNegative), followed by PRRSV infection. As shown in Figures 3(a) and 3(b), knockdown of TLR4 significantly decreased PRRSV-induced IL-1*β* mRNA expression and secretion, while no appreciable change was observed in cells transfected with siRNA targeting other tested receptor molecules compared to cells transfected with psiNegative. These results suggest that TLR4 may contribute to PRRSV-induced IL-1*β* production. Because LPS is the agonist of TLR4, before drawing this conclusion, we have to rule out the possibility that a contaminating LPS in virus stocks may be involved in these responses. To this end, we first measured the level of LPS in virus stocks by Limulus assay and no detectable amount of LPS was observed. Chemical polymyxin B (PMB) can bind lipid A domain of LPS and is always used to inhibit LPS-mediated inflammatory response [32]. Thus, we tested the effect of PMB on PRRSV-induced IL-1*β* production. As shown in Figure 4(a), treatment with 20 *μ*g/mL of PMB did not significantly affect PRRSV proliferation. The mRNA expression and secretion of IL-1*β* induced by PRRSV infection were also not inhibited by PMB, while the same treatment significantly abrogated the IL-1*β* production by LPS (Figures 4(b) and 4(c)). Together, these results indicated that the TLR4 signaling is really involved in PRRSV-induced IL-1*β* production.

It is well known that two adaptor molecules, TRIF and MyD88, are recruited by TLR4 to mediate its downstream signaling [33]. To further identify which adaptor is utilized by PRRSV to induce IL-1*β* production, we also used siRNA to knock down endogenous expression of MyD88 or TRIF as described previously [34]. As shown in Figures 3(c) and 3(d), compared to the negative control siRNA, knockdown of MyD88 significantly reduced PRRSV-induced IL-1*β* mRNA expression and secretion in PAMs but no appreciable change after knockdown of TRIF. These results indicated that MyD88 is the key adaptor downstream of TLR4 responsible for PRRSV-induced IL-1*β* production.

### 3.4. NF-*κ*B, ERK1/2, and p38 Mediate PRRSV-Induced IL-1*β*

**Figure 5:**
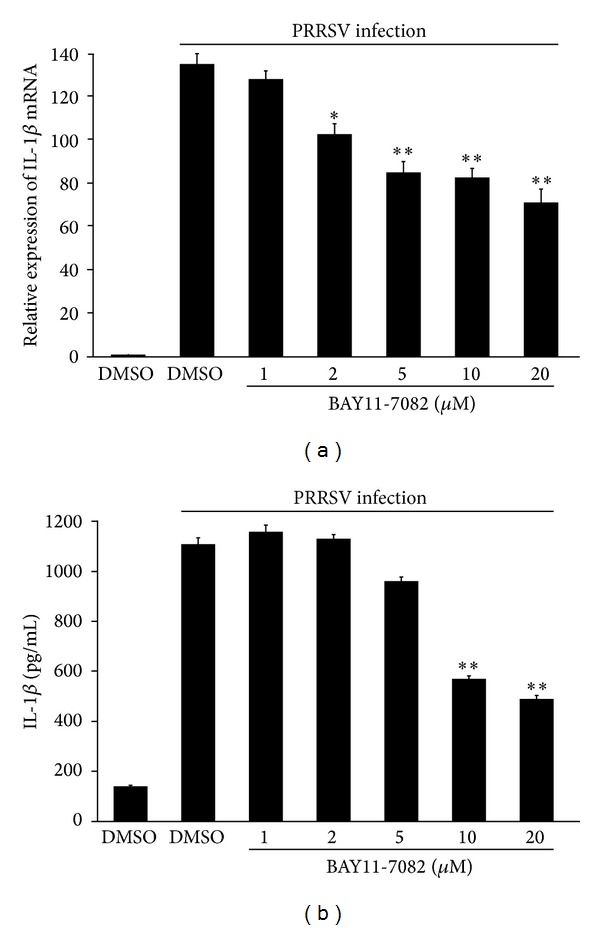
NF-*κ*B is required for PRRSV-induced IL-1*β* production. (a, b) PAMs were mock infected or infected with PRSSV at a MOI of 0.1, followed by treatment with NF-*κ*B inhibitor (1, 2, 5, 10, and 20 *μ*M) or DMSO vehicle in the absence of serum, for 36 h. The cells and supernatants were then harvested separately and analyzed by real-time RT-PCR (a) and ELISA (b), respectively. *P < 0.05 and **P < 0.01 compared with DMSO-treated cells plus PRRSV infection.

The downstream signaling of all TLR receptors involves three major signaling pathways: MAPKs, interferon regulatory factors (IRFs), and NF-*κ*B [35]. Previous studies revealed that PRRSV infection inhibited activation of IRFs [36–41], while signaling of NF-*κ*B and MAPK was activated [39, 42–45]. Thus, we further investigated the role of NF-*κ*B and MAPKs in PRRSV-induced IL-1*β* production in PAMs. To this end, PAMs were infected with PRRSV, followed by treatment with a specific NF-*κ*B inhibitor (BAY11-7082) at different doses (1, 2, 5, 10, and 20 *μ*M). As shown in Figures 5(a) and 5(b), cells treated with BAY11-7082 exhibited a decreased ability to upregulate IL-1*β* mRNA expression and protein secretion in a dose-dependent manner after PRRSV infection, indicating that NF-*κ*B is required for IL-1*β* production during PRRSV infection.

**Figure 6:**
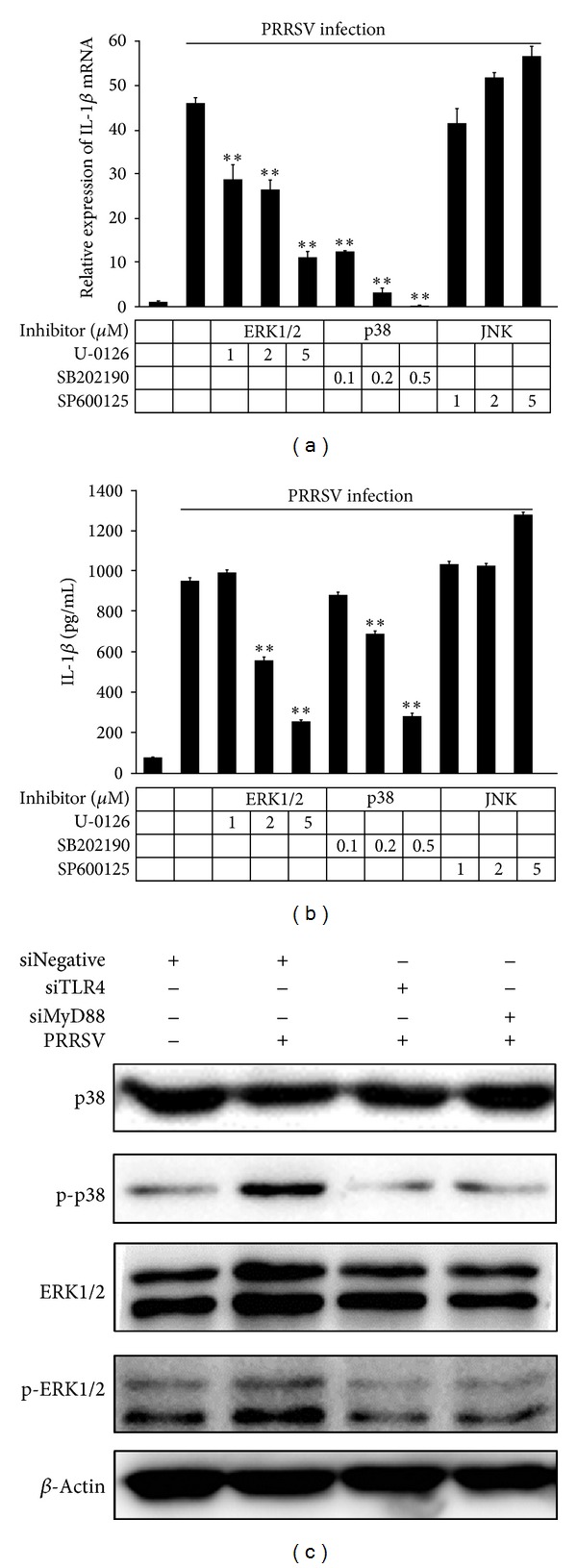
MAPK p38 and ERK1/2 are critical for PRRSV-induced IL-1*β* production in PAMs. Experiments were performed similar to those described in Figure 3 except that ERK1/2 inhibitor (1, 2, and 5 *μ*M), p38 MAPK inhibitor (0.1, 0.2, and 0.5 *μ*M), or JNK inhibitor (1, 2, and 5 *μ*M) was used. Cells and supernatants were then harvested and analyzed by IL-1*β*-specific real-time RT-PCR (a) and ELISA (b), respectively. **P < 0.01 compared with DMSO plus PRRSV infection.

Previous studies have demonstrated that PRRSV could activate p38 MAPK and ERK1/2 signaling [42, 44], and p38 MAPK and ERK1/2 are downstream signaling of TLR4 receptors [35]. To examine the role of the MAPK signaling cascade in the regulation of IL-1*β* during PRRSV infection, PAMs were treated with SB202190, U-0126, and SP600125, specific inhibitors of MAPK p38, ERK1/2, and JNK, respectively, at different doses. As shown in Figures 6(a) and 6(b), treatment with the JNK inhibitor SP600125 had no effect on IL-1*β* expression in PRRSV-infected PAMs, but the IL-1*β* mRNA and protein expression were reduced following a dose-dependent increase of the ERK1/2 inhibitor U-0126 and the p38 inhibitor SB202190, indicating that the MAPK p38 and ERK1/2 appear to be involved in PRRSV-induced IL-1*β* production. To further confirm these results, we detected the p38 MAPK and ERK1/2 phosphorylation. As shown in Figure 6(c), knockdown of TLR4 and MyD88 could inhibit PRRSV-induced p38 MAPK and ERK1/2 phosphorylation. These results further confirmed that the ERK1/2 and p38 are involved in PRRSV-induced IL-1*β* production.

### 3.5. The NLRP3 Inflammasome Is Required for PRRSV-Mediated IL-1*β* Maturation and Secretion

**Figure 7:**
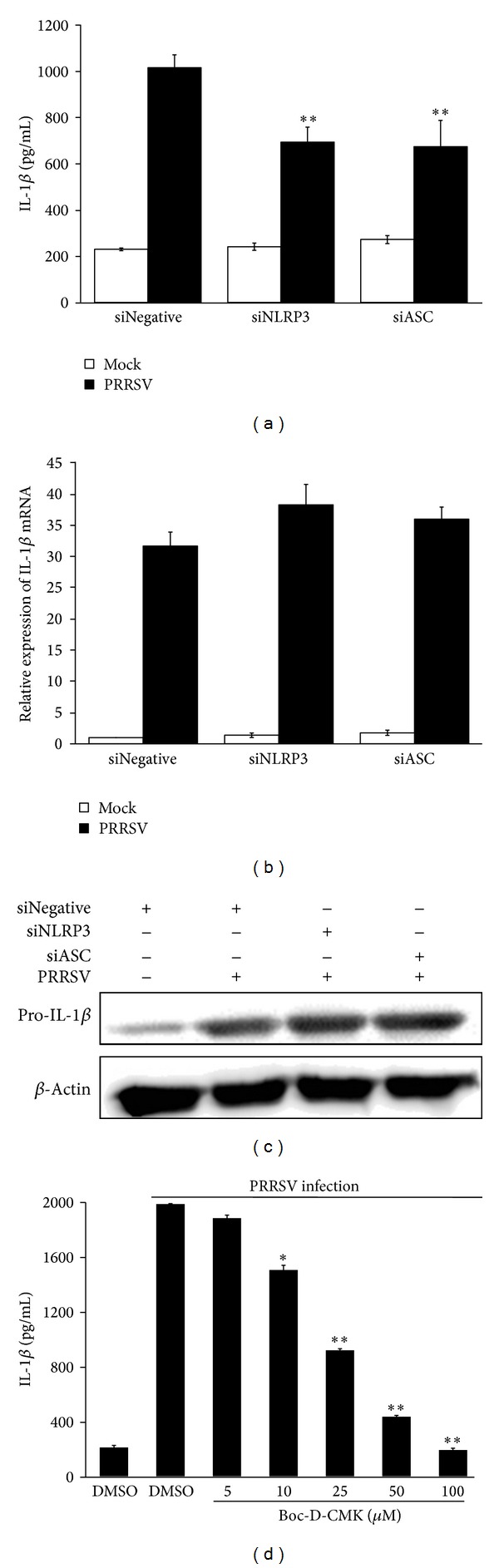
PRRSV-induced IL-1*β* secretion requires NLRP3 inflammasome. (a–c) PAMs were transfected with 80 nM of psiNegative, psiNLRP3, or psiASC, respectively. At 24 h posttransfection, cells were mock infected or infected with PRRSV at a MOI of 0.1. The supernatants and cells were harvested separately 36 h later and analyzed by IL-1*β* ELISA (a) and real-time RT-PCR (b), respectively. For Western blotting to detect pro-IL-1*β*, cells were harvested at 24 h postinfection by using polyclonal antibody against pro-IL-1*β* (c). (d) PAMs were mock infected or infected with PRRSV at a MOI of 0.1, followed by treatment with caspase 1 inhibitor (5, 10, 25, 50, and 100 *μ*M) or DMSO vehicle in the absence of serum, for 36 h. The supernatants were then harvested and analyzed by IL-1*β*-specific ELISA. *P < 0.05 and **P < 0.01 compared with DMSO-treated cells plus PRRSV infection.

The second signal for IL-1*β* production is the inflammasome complex assembly and subsequent caspase-1 activation [3, 5]. The NLRP3 inflammasome is the most characterized inflammasome complex and involved in IL-1*β* maturation during infections with several viruses [46]. To investigate the role of the NLRP3 inflammasome in PRRSV-mediated production of IL-1*β*, specific siRNAs targeting the porcine NLRP3 inflammasome receptor (NLRP3) and adaptor (ASC) were designed (Table  S1) (Supplementary Materials). The reduced expression levels of NLRP3 and ASC in PAMs treated with specific siRNAs were confirmed by real-time PCR (Figures  S1K and L) (Supplementary Materials). Silencing NLRP3 or ASC in PAMs significantly decreased IL-1*β* secretion following PRRSV infection (Figure 7(a)), while no appreciable change was observed in IL-1*β* mRNA (Figure 7(b)) and pro-IL-1*β* protein expression (Figure 7(c)) compared to cells transfected with psiNegative.

To further investigate whether caspase 1 activation is required for IL-1*β* release during PRRSV infection, PAMs were infected with PRRSV, followed by treatment with a specific caspase 1 inhibitor (Boc-D-CMK) at different doses (5, 10, 25, 50, and 100 *μ*M). As shown in Figure 7(d), cells treated with Boc-D-CMK exhibited a decreased ability to upregulate IL-1*β* production in a dose-dependent manner after PRRSV infection. Based on these results, we concluded that NLRP3/ASC/caspase 1 inflammasome is indispensable for efficient IL-1*β* secretion but not for its mRNA expression during PRRSV infection.

### 3.6. TLR4/MyD88/NF-*κ*B Signaling Pathway Is Involved in PRRSV-Induced Expression of NLRP3 Inflammasome Components

**Figure 8:**
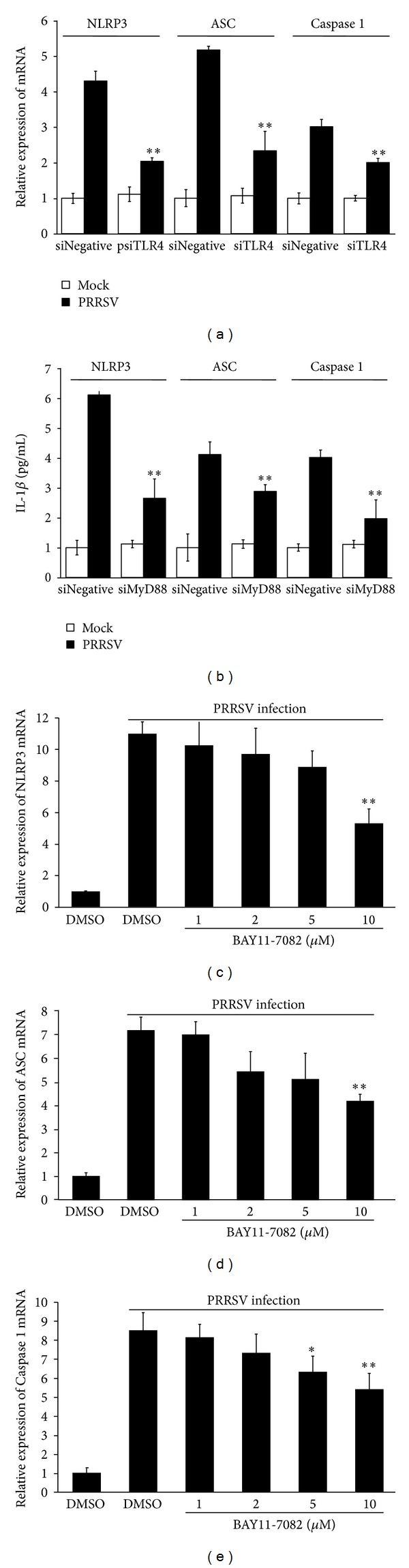
TLR4/MyD88/NF-*κ*B signaling pathway is involved in PRRSV-induced expression of NLRP3 inflammasome components. (a, b) PAMs were transfected with 80 nM of psiNegative, psiTLR4, or psiMyD88, respectively. At 24 h posttransfection, cells were mock infected or infected with PRRSV at a MOI of 0.1. The cells and supernatants were harvested separately 36 h later and analyzed by NLRP3/ASC/caspase 1 specific real-time RT-PCR. (c–e) PAMs were mock infected or infected with PRSSV at a MOI of 0.1, followed by treatment with NF-*κ*B inhibitor (1, 2, 5, and 10 *μ*M) or DMSO vehicle in the absence of serum, for 36 h. The cells were then harvested to analyse the mRNA expression of NLRP3 (c), ASC (d), and caspase 1 (e), respectively, by real-time RT-PCR. *P < 0.05 and **P < 0.01 compared with DMSO-treated cells plus PRRSV infection.

Having deciphered the signaling pathways involved in PRRSV-induced IL-1*β* production, we further evaluated the relationship between TLR4/MyD88/NF-*κ*B signaling and NLRP3 inflammasome. Because no antibody against porcine NLRP3, ASC, and caspase 1 can be available, we analyzed the mRNA expression of these molecules after interfering with specific siRNA or inhibiting with specific inhibitors for TLR4/MyD88/NF-*κ*B signaling. As shown in Figures 8(a) and 8(b), knockdown of TLR4 and MyD88 by siRNA significantly decreased PRRSV-induced mRNA expression of NLRP3, ASC, and caspase 1. Similar results could be observed when PAMs were treated with NF-*κ*B-specific inhibitor BAY11-7082 after PRRSV infection (Figures 8(c)–8(e)). Based on these data, we concluded that TLR4/MyD88/NF-*κ*B signaling pathway is involved in PRRSV-induced expression of NLRP3, ASC, and caspase 1.

## 4. Discussion

Interstitial pneumonia is a condition characteristic in pigs after PRRSV infection [18]. More recently, Morgan et al. compared the pathogenesis of three different European PRRSV strains and proposed that the increased clinical and pathological effect of the highly pathogenic PRRSV strain is more likely to be caused by an enhanced inflammatory immune response rather than higher levels of virus replication [47]. Similarly, previous study also demonstrated that the highly pathogenic North American type PRRSV, which emerged in China and South East Asia, induced stronger proinflammatory responses than the classical North American type PRRSV [20]. These observations highlight the important role of inflammatory response in infection and pathogenesis of PRRSV. Thus, analyses of the underlying mechanisms responsible for inflammatory responses may contribute to a deeper understanding of the infection and pathogenesis of PRRSV. IL-1*β* is a key proinflammatory cytokine and plays a very important role in shaping the inflammatory response against pathogens [2]. In this study, we investigated the mechanisms, particularly the cellular sensors and signaling pathways, responsible for the transcription and secretion of IL-1*β* in response to highly pathogenic PRRSV infection in primary PAMs, the target cells of PRRSV infection* in vivo*. Our results clearly showed that PRRSV infection significantly induced IL-1*β* production and processing in primary PAMs in a manner that was dependent on TLR4/MyD88 signaling and NLRP3 inflammasome activation, respectively.

The production and release of IL-1*β* are tightly regulated at several levels: the transcription of the gene and synthesis of immature pro-IL-1*β* protein, the proteolytic processing/cleavage of pro-IL-1*β* into the mature form of IL-1*β*, and secretion of mature IL-1*β* into the extracellular milieu [2, 3]. IL-1*β* gene transcription and translation are triggered by most viruses through a variety of PRRs, transcription factors, and cytoplasmic signals [3, 48]. In this study, we for the first time identified TLR4 as the key receptor and MyD88 as the key adaptor to mediate PRRSV-induced IL-1*β* transcription. TLR4 mainly recognizes LPS derived from the outer membrane of Gram-negative bacteria. However, TLR4 is also involved in the recognition of viral envelope proteins, including those of RSV, vesicular stomatitis virus, Ebola virus, and mouse mammary tumor virus [49–53]. Ebola virus glycoprotein interacts with TLR4 to induce proinflammatory cytokines [51]. RSV F protein is a TLR4 agonist and activates the innate immune response via TLR4 [52]. Furthermore, infection with RSV results in increased expression of TLR4 mRNA, protein, and increased TLR4 membrane localization [49]. Interestingly, PRRSV infection also induced TLR4 mRNA expression in the brain and respiratory tract of pigs [54]. Thus, it is possible that TLR4 senses one or more proteins encoded by PRRSV to induce an inflammatory response. Identifying the PRRSV protein(s) that binds to TLR4 is of interest and requires further investigation.

In this study, we also demonstrated that NF-*κ*B, p38, and ERK1/2 were required for PRRSV-induced IL-1*β* transcription and secretion. These results are expected since both NF-*κ*B and MAPK are downstream molecules in the TLR/MyD88 signaling pathway. It has also been reported that PRRSV infection could induce the activation of the NF-*κ*B signaling pathway [43]; however, the responsible TLR triggered by PRRSV to activate NF-*κ*B signaling had not been identified. Based on our results, we speculate that PRRSV induces the activation of both NF-*κ*B signaling and IL-1*β* production via TLR4/MyD88 signaling. Although MAPK has been demonstrated to be involved in virus-induced IL-1*β* production, different MAPKs were utilized by different viruses. For example, herpes simplex virus 1 has been reported to induce IL-1*β* production through the p38 MAPK signaling pathway, while ERK1/2 and JNK signaling pathways were utilized by HIV TAT protein to activate the transcription of IL-1*β* [55, 56]. Previous studies have demonstrated that PRRSV infection could activate MAPK p38, ERK1/2, and JNK in PAMs and Marc-145 cells [42, 44, 45]. However, only ERK1/2 and p38 MAPK pathways were associated with PRRSV-induced IL-1*β* expression in our present study, and TLR4/MyD88 signaling plays an important role in ERK1/2 and p38 phosphorylation. Based on this result and combined with the role of TLR4/MyD88/NF-*κ*B in IL-1*β* expression, we conclude that the TLR4/MyD88 pathway and its downstream signaling molecules NF-*κ*B, p38, and ERK1/2 were required for PRRSV-induced IL-1*β* production.

The proteolytic processing of pro-IL-1*β* is mediated by the inflammasome complex. At least nine inflammasome complexes have been described to date [57]. In addition to NLR inflammasomes (NLRP1, NLRP3, NLRP6, NLRP12, and NLRC4), there are four additional inflammasomes (AIM2, RIG-I, IFI6, and PYRIN) which form by non-NLR sensor proteins [57]. Work over the last years has identified the NLRP3 and RIG-I inflammasomes as key regulators of RNA virus-induced IL-1*β* production [8, 57]. The NLRP3 inflammasome is well characterized and many viruses such as adenovirus, influenza A virus, HIV, encephalomyocarditis virus, and RSV are known to promote IL-1*β* production by activating the NLRP3 inflammasome [13, 58–60]. The RIG-I inflammasome has dual functions in inducing IL-1*β* production [61–63]. The first function is to sense cytosolic viral infection and activate NF-*κ*B via MAVS and a complex of the adaptor CARD9 and Bcl-10, resulting pro-IL-1*β* production; the second function is to bind ASC and thereby trigger caspase-1-dependent inflammasome activation and IL-1*β* generation via a NLRP3-independent mechanism [61, 63]. However, the dual functions of RIG-I do not appear to play a role in PRRSV-induced IL-1*β* production because neither pro-IL-1*β* mRNA expression nor mature IL-1*β* secretion was influenced during PRRSV infection in RIG-I knock-down cells. This result is consistent with our previous study in which we demonstrated that PRRSV infection interfered with the RIG-I signaling pathway [39]. Unlike the RIG-I, the NLRP3 inflammasome is required for PRRSV-induced IL-1*β* production. However, the NLRP3 inflammasome only contributed to IL-1*β* secretion but not to its transcription during PRRSV infection. It is uncertain, whether other inflammasomes also function in the secretion of IL-1*β* in PRRSV-infected cells; thus this possibility cannot be excluded and warrant further study.

Until now, the precise mechanism that initiates activation of the NLRP3 inflammasome and the subsequent activation of caspase 1 is not fully understood. By analyzing the relationship of NLRP3 expression and NLRP3 inflammasome activation, Bauernfeind et al. found that NLRP3 expression level is a limiting factor for NLRP3 inflammasome activation and NLRP3 expression is tightly regulated by TLR-mediated NF-*κ*B signal [64]. Indeed, there exist NF-*κ*B and AP1 binding sites in the NLRP3 promoter region [13, 65]. Thus, crosstalks between TLRs and NLRP3 inflammasome have been proposed to be essential for the fine regulation of virus-induced IL-1*β* production. A body of evidence implicates that TLRs and NF-*κ*B play critical role in the priming activation of NLRP3 inflammasome [13, 57, 65, 66]. TLR4 agonist significantly upregulated NLRP3 expression via a NF-*κ*B dependent manner in murine macrophages [67]; NLRP3 expression was significantly increased via TLR2/MyD88/NF-*κ*B signaling during RSV infection [13]. Because the antibody against porcine NLRP3 is not available, we did not investigate the NLRP3 protein expression in PRRSV-infected PAMs. However, our data showed that PRRSV infection significantly upregulated mRNA expressions of NLRP3 and ASC, as well as caspase 1. Furthermore, knockdown of TLR4 or MyD88 or inhibition with NF-*κ*B-specific inhibitor also decreased expressions of NLRP3, ASC, and caspase 1 after PRRSV infection. Thus, TLR4/MyD88/NF-*κ*B signaling pathway is involved in PRRSV-induced pro-IL-1*β* and NLRP3 expression (signal 1), and NLRP3 inflammasome activation is involved in PRRSV-induced IL-1*β* maturation and secretion (signal 2).

## 5. Conclusion

To conclude, we have uncovered the pathways involved in the recognition of PRRSV to the production and release of IL-1*β*. PRRSV-induced IL-1*β* production is tightly regulated at the levels of transcription, translation, and posttranslational processing. The TLR4/MyD88 pathway and its downstream signaling molecules (NF-*κ*B, p38, and ERK1/2) play major roles in PRRSV-induced IL-1*β* mRNA expression and pro-IL-1*β* production, while the NLRP3 inflammasome is required for the processing of pro-IL-1*β* and pro-IL-1*β* secretion. During preparing this paper, Zhang et al. reported that ectopic expression of PRRSV-encoded small envelope protein E, an ion channel-like protein, triggers the activation of inflammasomes [68]. Whether or not other PRRSV-encoded proteins are involved in inflammasome activation and IL-1*β* production and which inflammasome(s) is activated by PRRSV or its encoded protein(s) remain undetermined. Dissection of these issues is important for better understanding of PRRSV-induced inflammation responses.

## Supplementary Material

Supplementary table 1: The siRNAs used in this study.
Supplementary table 2: The primers used in this study for real-time RT-PCR.
Supplementary figure 1: Knockdown efficiency of siRNAs against RIG-I, Toll-like receptors, NLRP3 and ASC. PAMs were transfected with 80 nM of psiRIG-I, psiTLR1-9, psiNLRP3 and psiASC, respectively. Cells transfected with psiNegative were used as controls. The transfected cells were collected at 24 h post-transfection to examine the endogenous transcription of porcine RIG-I, TLR1-9, NLRP3 and ASC mRNA by real-time RT-PCR. The expression level of RIG-I, TLR1-9, NLRP3 and ASC were first normalized to that of *β*-actin in the same sample and then compared with that of cells transfected with psiNegative. The presented data indicates the mRNA expression level of the tested molecules in cells transfected with specific siRNA relative to that in psiNegative-transfected cells. ∗∗P < 0.01 compared with cells transfected with psiNegative.
