# Supplementary material for: Porcine Reproductive and Respiratory Syndrome Virus Induces IL-1β Production Depending on TLR4/MyD88 Pathway and NLRP3 Inflammasome in Primary Porcine Alveolar Macrophages
Source: Mediators Inflamm. 2014 May 21;2014:403515. doi: 10.1155/2014/403515 (PMC4055429; doi:10.1155/2014/403515)
Supplement: Supplementary file 1 — Supplementary table 1: The siRNAs used in this study. Supplementary table 2: The primers used in this study for real-time RT-PCR. Supplementary figure 1: Knockdown efficiency of siRNAs against RIG-I, Toll-like receptors, NLRP3 and ASC. PAMs were transfected with 80 nM of psiRIG-I, psiTLR1-9, psiNLRP3 and psiASC, respectively. Cells transfected with psiNegative were used as controls. The transfected cells were collected at 24 h post-transfection to examine the endogenous transcription of porcine RIG-I, TLR1-9, NLRP3 and ASC mRNA by real-time RT-PCR. The expression level of RIG-I, TLR1-9, NLRP3 and ASC were first normalized to that of β-actin in the same sample and then compared with that of cells transfected with psiNegative. The presented data indicates the mRNA expression level of the tested molecules in cells transfected with specific siRNA relative to that in psiNegative-transfected cells. ∗∗P < 0.01 compared with cells transfected with psiNegative. [file 403515.f1.pdf]

Supplementary Materials

Table S1. The siRNAs used in this study.

Table S2. The primers used in this study for real-time RT-PCR.

Figure S1. Knockdown efficiency of siRNAs against RIG-I, Toll-like receptors, NLRP3 and ASC. (A-L) PAMs were transfected with 80 nM of psiRIG-I, psiTLR1-9, psiNLRP3 and psiASC, respectively. Cells transfected with psiNegative were used as controls. The transfected cells were collected at 24 h post-transfection to examine the endogenous transcription of porcine RIG-I, TLR1-9, NLRP3 and ASC mRNA by real-time RT-PCR. The expression level of RIG-I, TLR1-9, NLRP3 and ASC were first normalized to that of β-actin in the same sample and then compared with that of cells transfected with psiNegative. The presented data indicates the mRNA expression level of the tested molecules in cells transfected with specific siRNA relative to that in psiNegative-transfected cells. **P < 0.01 compared with cells transfected with psiNegative.

Table S1. The siRNAs used in this study.

Gene name	Sequence region	siRNA sequence (Sense)	siRNA sequence (Anti-sense)
RIG-I	650-670	5’-GGU ACA AAG UUG CAG GCA UTT -3’	5’-AUG CCU GCA ACU UUG UAC CTT-3’
TLR1	1160-1180	5’-CCC ACA AAG UUA CAU CUA UTT-3’	5’-AUA GAU GUA ACU UUG UGG GTT-3’
TLR2	1247-1267	5’-GCC CUU CCU ACA CAC UUU ATT-3’	5’-UAA AGU GUG UAG GAA GGG CTT-3’
TLR3	1795-1815	5’-GGU CUU GAG AAA CUA CAA ATT-3’	5’-UUU GUA GUU UCU CAA GAC CTT-3’
TLR4	1947-1967	5’-CAG GAA UCC UGG UCU AUA ATT-3’	5’-UUA UAG ACC AGG AUU CCU GTT-3’
TLR5	953-973	5’-GCC UUC AAC AAG AUA AAC ATT-3’	5’-UGU UUA UCU UGU UGA AGG CTT-3’
TLR6	820-840	5’-GCC CAA ACC UGU AGA AUA UTT-3’	5’-AUA UUC UAC AGG UUU GGG CTT-3’
TLR7	342-362	5’-CCA GAA GGC AGC AGA UUA ATT -3’	5’-UUA AUC UGC UGC CUU CUG GTT-3’
TLR8	786-806	5’-GCC AGA AUC UUU GAG AGA ATT-3’	5’-UUC UCU CAA AGA UUC UGG CTT-3’
TLR9	1074-1094	5’-CCU UCA AUU ACC ACA AGA ATT-3’	5’-UUC UUG UGG UAA UUG AAG GTT-3’
NLRP3	3003-3023	5’-GCC UUA AGU UGU GUG AAA UTT-3’	5’-AUU UCA CAC AAC UUA AGG CTT-3’
ASC	310-330	5’-CCU GAA AGC AGA CAA CAA ATT-3’	5’-UUU GUU GUC UGC UUU CAG GTT-3’
Negative control		5’-UUC UCC GAA CGU GUC ACG UTT-3’	5’-ACG UGA CAC GUU CGG AGA ATT-3’

Table S2. The primers used in this study for real-time RT-PCR.

Gene name	Forward primer sequence	Reverse primer sequence
β-actin	5’-TGAGAACAGCTGCATCCACTT-3’	5’-CGAAGGCAGCTCGGAGTT-3’
IL-1β	5’-TCCACTTGGCGGTGATCA-3’	5’-TCCACTTGGCGGTGATCA-3’
RIG-I	5’- AGAGCAGCGGCGGAATC-3’	5’- GGCCATGTAGCTCAGGATGAA-3’
TLR1	5’-CCATCCCCAGCAGCTATCA-3’	5’-CCTTGGGCCATTCCAAATAA-3’
TLR2	5’-TTCTCCCACTTCCGTCTCTTTG-3’	5’-TCTTCTTCTCGATGGGTTCCA-3’
TLR3	5’-CATTGAGAATCTATCCCTGAGCAA-3’	5’-TGAGGTTTGTCTGCTTTAGTCCAA-3’
TLR4	5’-CCTGCCTGTGCTGAGTTTCA-3’	5’-AAGGTGAGAACTGACGCACTAATG-3’
TLR5	5’-CTCCTTTTTAAGCCTTGCGGATA-3’	5’-TAGCATTTCCAAGGCCATGTT-3’
TLR6	5’-CACCTCTCTGACATCAGCTTTCTC-3’	5’-GATATCAAGGCACTGCATCCTATTC-3’
TLR7	5’-AAACTCTGCCCTGTGATGTCAGT-3’	5’-GAATCGCTGTCAAGTGCTTGTC-3’
TLR8	5’-AAAAAGCACGTCCCTGAAAGAA-3’	5’-TACCTGTCATCTTGGGCATTCC-3’
TLR9	5’-TGTCCAGCCTACGAACTCTCAA-3’	5’-TTGGGCTCGATGGTCATGT-3’
NLRP3	5’-AGCAGATTCCAGTGCATCAAAG-3’	5’-CCTGGTGAAGCGTTTGTTGAG-3’
ASC	5’-GAGCCAAGCCAGCTGGAA-3’	5’-CGAAGTGCAGTGCTGGTTTG3’
Caspase-1	5’-TGGTGCTCATGTCTCATGGAA-3’	5’-ACATCTGGGACTTCTTCGGAGTAT-3’

Figure S1.

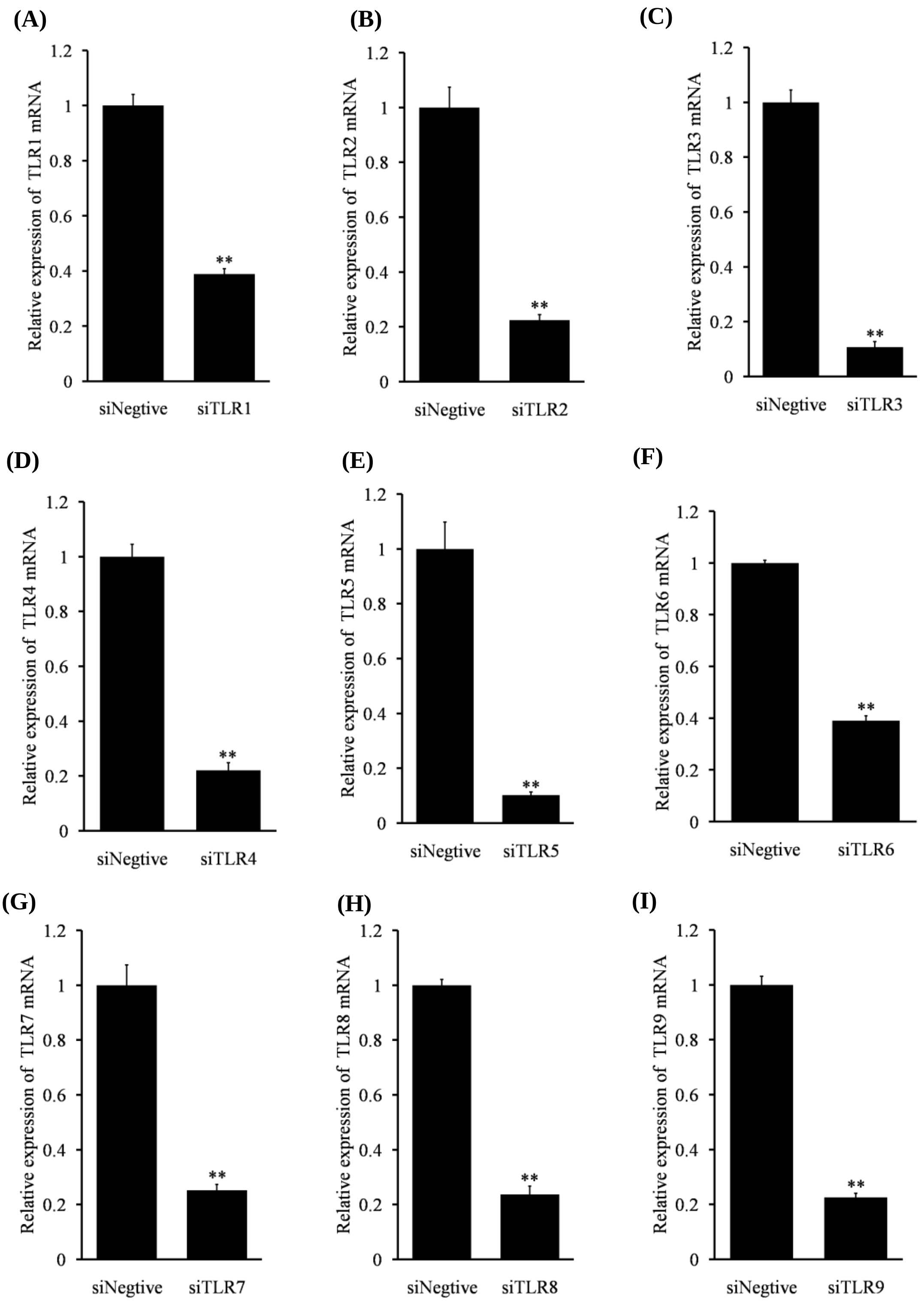

(A)
Relative expression of TLR1 mRNA
siNegtive
siTLR1
**
(B)
Relative expression of TLR2 mRNA
siNegtive
siTLR2
**
(C)
Relative expression of TLR3 mRNA
siNegtive
siTLR3
**
(D)
Relative expression of TLR4 mRNA
siNegtive
siTLR4
**
(E)
Relative expression of TLR5 mRNA
siNegtive
siTLR5
**
(F)
Relative expression of TLR6 mRNA
siNegtive
siTLR6
**
(G)
Relative expression of TLR7 mRNA
siNegtive
siTLR7
**
(H)
Relative expression of TLR8 mRNA
siNegtive
siTLR8
**
(I)
Relative expression of TLR9 mRNA
siNegtive
siTLR9
**
0
0.2
0.4
0.6
0.8
1
1.2

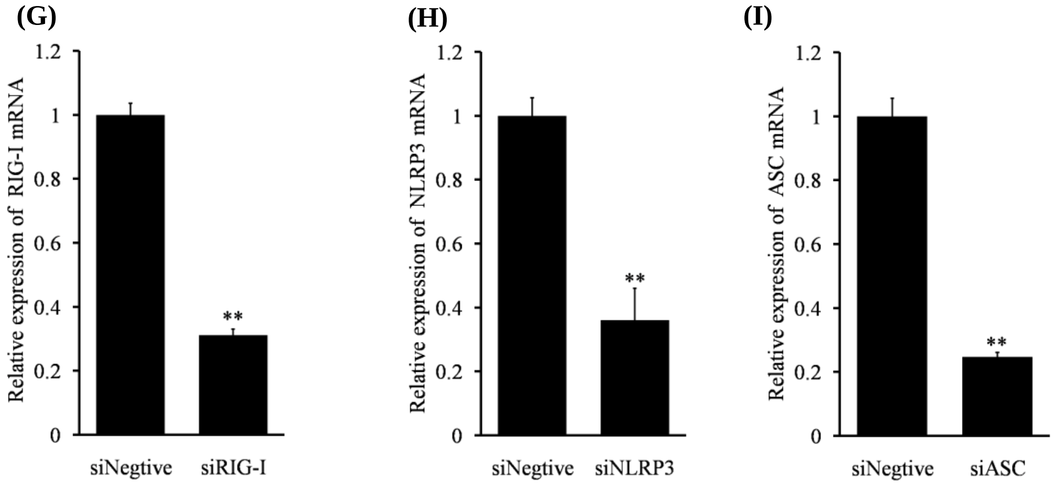

(G)
(H)
(I)
Relative expression of RIG-I mRNA
Relative expression of NLRP3 mRNA
Relative expression of ASC mRNA
1.2
1
0.8
0.6
0.4
0.2
0
**
siNegtive
siRIG-I
siNLRP3
siASC
